# A GntR Family Transcription Factor (VPA1701) for Swarming Motility and Colonization of *Vibrio parahaemolyticus*

**DOI:** 10.3390/pathogens8040235

**Published:** 2019-11-13

**Authors:** Dan Gu, Hongmei Meng, Yang Li, Haojie Ge, Xinan Jiao

**Affiliations:** 1 Jiangsu Co-Innovation Center for Prevention and Control of Important Animal Infectious Diseases and Zoonoses, Yangzhou University, Yangzhou 225009, China; 006491@yzu.edu.cn (D.G.); MX120170784@yzu.edu.cn (H.M.); MZ120171036@yzu.edu.cn (Y.L.); mz120171057@yzu.edu.cn (H.G.); 2 Jiangsu Key Laboratory of Zoonosis, Yangzhou University, Yangzhou 225009, China; 3 Key Laboratory of Prevention and Control of Biological Hazard Factors (Animal Origin) for Agrifood Safety and Quality, Ministry of Agriculture of China, Yangzhou University, Yangzhou 225009, China; 4 Joint International Research Laboratory of Agriculture and Agri-Product Safety of the Ministry of Education, Yangzhou University, Yangzhou 225009, China

**Keywords:** *Vibrio parahaemolyticus*, VPA1701, CalR, lateral flagellar system, swarming motility

## Abstract

Motility is important for virulence, biofilm formation, and the environmental adaptation of many bacteria. *Vibrio parahaemolyticus* (*V. parahaemolyticus*) contains two flagellar systems that are responsible for motility, and are tightly regulated by transcription regulators and sigma factors. In this study, we identified a novel transcription factor, VPA1701, which regulates the swarming motility of *V. parahaemolyticus*. The VPA1701 deletion mutant (Δ*VPA1701*) eliminated the swarming motility on the surface of BHI agar plates and reduced colonization in infant rabbits. RNA-seq assays, confirmed by qRT-PCR, indicated that VPA1701 regulated the expression of lateral flagellar cluster genes. Further analyses revealed that VPA1701 directly binds to the promoter region of the *flgBCDEFGHIJKL* cluster to regulate the expression of lateral flagellar genes. CalR was originally identified as a repressor for the swarming motility of *V. parahaemolyticus,* and it was inhibited by calcium. In this study, we found that VPA1701 could inhibit the expression of the *calR* gene to increase the swarming motility of *V. parahaemolyticus*. Calcium downregulated the expression of *calR*, indicating that calcium could increase swarming motility of Δ*VPA1701* by inhibiting *calR*. Thus, this study illustrates how the transcription factor VPA1701 regulates the expression of lateral flagellar genes and *calR* to control the swarming motility of *V. parahaemolyticus*.

## 1. Introduction

*V. parahaemolyticus* is a foodborne pathogen sometimes found in contaminated seafood, which can cause acute-gastroenteritis and hemorrhagic sepsis in humans [1,2]. The major virulence factors of *V. parahaemolyticus* are hemolysin (thermostable direct hemolysin [TDH], TDH-related hemolysin [TRH]), type III secretion systems (T3SS1 and T3SS2), type VI secretion systems (T6SS1 and T6SS2), biofilm formation, motility, protease production and iron uptake system [3,4,5,6,7,8]. The pathogenesis processes of *V. parahaemolyticus* include motility, adhesion, invasion, proliferation, production of toxins in vivo, and the damage to cells and tissues [9,10]. Motility plays an essential role in the infection of *V. parahaemolyticus* in the host, and the protease production can also benefit host invasion. The *V. parahaemolyticus* contains two flagellar systems: polar flagellum and lateral flagellum. The polar flagellum is encoded by the polar flagellar genes responsible for the swimming motility, and the lateral flagellum is responsible for the swarming motility encoded by lateral flagellar genes [11]. A previous study also indicated that *V. parahaemolyticus* lacking flagellum could not adhere to either normal or degenerating tissue cells [12]. Therefore, the flagellum may help bacteria to adhere to the surface of cells and promote the biofilm formation that contributes to the pathogenicity of bacteria [13,14].

Motility provides a more favorable living condition for microorganisms, and 60% of bacteria contain the flagellar systems responsible for motility. These flagellar systems can be divided into three types: the polar flagellar system (*Vibrio cholerae*), the lateral flagellar system (*Salmonella enterica*), and the dual flagellar systems (*V. parahaemolyticus, Vibrio alginolyticus,* and *Aeromonas spp*) [14,15]. In *V. parahaemolyticus*, the dual flagellar systems play an essential role in complex environments, the sheathed polar flagellar system powered by MotAB and MotXY sodium pumps propels the bacterium in a liquid environment, while the lateral flagellar system motor remains to be identified [16,17]. The polar flagellar system is encoded by more than 60 genes located in chromosome I and regulated by FlaK, and the lateral flagellar system is encoded by 38 genes located in chromosome II and regulated by LafK. Additionally, the transcriptional regulator LafK may compensate for the function of FlaK to enable swimming ability in some bacteria [18]. 

The expression of flagellar genes is hierarchical, with specific transcription regulators and sigma factors controlling the early, middle, and late gene expression. The flagellar genes and specific regulators are similar in vibriosis [19]. The expression of polar flagellum genes is modulated by the master regulator FlaK, which is a σ^54^-dependent regulator and activates the expression of class II flagellar genes. Subsequently, the regulators FlaM and σ^54^ initiate the expression of class III genes. The expression of class II and class III genes is in the middle of the hierarchy. Lastly, the expression of class IV genes is directly activated by a σ^28^-dependent regulator [20]. In the expression of the lateral flagellar system, the master regulator LafK activates the class II genes, including *flgAMN*, *flgBCDEFGHIJ*, *fliDSTKLAmotAB,* and *motYlafKfliEFGHI,* early in the hierarchy. Then, the expression of class III genes, including *flgKL*, *lafA*, *fliDSTKLAmotAB,* and *flgMN,* are activated by σ^28^, which is encoded by *fliA* [14,20,21]. Thus, the expression of flagellar genes is tightly regulated by the specific regulators and sigma factors in a spatiotemporal-dependent way.

The motility is affected by various cellular processes, such as the quorum sensing system, type III secretion system (T3SS), signaling molecules, and transcription regulators. The quorum sensing system plays an essential role in the regulation of motility in *V. alginolyticus*, *V. harveyi,* and *V. parahaemolyticus* [22,23,24]. In *V. parahaemolyticus*, the T3SS1 transcription regulator ExsA inhibits the swarming motility by decreasing the expression of *lafK*, the master regulator of the lateral flagellar system [25]. The second messenger c-di-GMP directly binds to the flagellar master regulator FlrA and inhibits the motility of *Vibrio cholerae* [26]. Glucose regulates the synthesis of flagellum by inhibiting the polar localization of FapA in *V. vulnificus* [27]. Furthermore, a global transcriptional regulator, CalR, downregulates the expression of *laf* genes to inhibit the swarming motility of *V. parahaemolyticus* [25]. Additionally, ToxR acts as an activator of both swarming and swimming motility in *V. parahaemolyticus* [28]. 

The objective of this study was to assess the role of VPA1701 in the regulation of swarming motility in *V. parahaemolyticus*. A previous study used transposon-insertion sequencing (TIS) to identify 230 genes that contributed to the colonization of *V. parahaemolyticus* in infant rabbit small intestines [29]. In this study, we selected 22 of those genes to construct the deletion mutant strains and found that the colonization and swarming motility of Δ*VPA1701* had significantly decreased compared to the wild type. Furthermore, the VPA1701 directly bound to the promoter region of the flagellar genes to regulate the expression of lateral flagellar genes. Lastly, VPA1701 inhibited the expression of *calR* to control the swarming motility of *V. parahaemolyticus*. 

## 2. Results and Discussion 

### 2.1. Essential Roles of VPA1701 in the Colonization and Swarming Motility of V. parahaemolyticus

**Figure 1:**
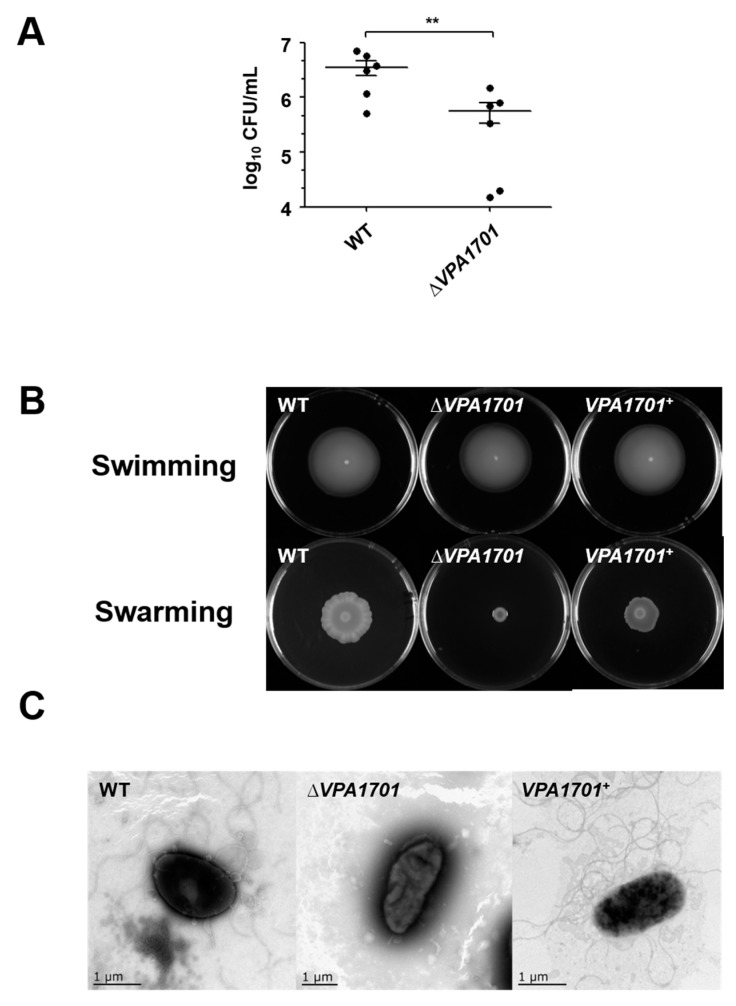
Essential roles of VPA1701 in the in vivo colonization and motility of *V. parahaemolyticus*. (**A**) The infant rabbit received WT and ∆*VPA1701* orogastrically, and the infant rabbit was killed to obtain the colonization data. The results are shown as the mean ± SD (n = 6); ** *p* < 0.05. (**B**) *V. parahaemolyticus* WT, ∆*VPA1701,* and *VPA1701^+^* were cultured in semi-solid plates (LB with 0.3% Agar) at 37 °C and solid plates (BHI with 1.5% Agar) at 30 °C to observe the swimming and swarming ability separately. (**C**) The generation of the lateral flagellum was observed by TEM of WT, ∆*VPA1701,* and *VPA1701^+^*.

VPA1701 has been identified as a transcriptional regulatory protein in the GntR family that contains a conserved helix-turn-helix (HTH) for DNA binding at the N-terminus and an effector binding domain involved in oligomerization at the C-terminus [30]. BlastP search against GenBank indicated that *V. parahaemolyticus* VPA1701 shared 98.86%, 98.48%, 92.72%, and 89.27% identities to the homologous proteins in *Vibrio harveyi*, *Vibrio campbellii*, *Vibrio vulnificus,* and *Vibrio cholerae*, respectively (Supplementary Figure S1). The previous study used TIS to demonstrate that VPA1701 was involved in the colonization of *V. parahaemolyticus* in the intestines of infant rabbits [29]. In this study, we constructed the VPA1701 deletion mutant and complemented strains to confirm the function of VPA1701 in *V. parahaemolyticus*. Infant rabbits received a dose of the bacteria, delivered orogastrically, and observations showed that the colonization of *V. parahaemolyticus* was significantly reduced by ∆*VPA1701* compared to the WT (Figure 1A). 

During colonization and infection, the bacteria have evolved several mechanisms to control the expression of genes to enable them to adapt to different environments. This colonization ability was essential for the infection of *V. parahaemolyticus*, and the previous study has found that motility was important for the colonization and infection of *V. parahaemolyticus* and *Vibrio coralliilyticus* [9,31]. The quorum sensing regulator SwrT could inhibit a GntR family protein SwrZ to control the expression of *laf* genes and regulate the swarming motility of *V. parahaemolyticus* [32]. Therefore, the motility of WT, ∆*VPA1701,* and complemented strains were measured, and the results showed that there was no significant difference in swimming motility between WT and ∆*VPA1701* (Figure 1B). However, the swarming motility of ∆*VPA1701* was significantly decreased compared to the WT, and the swarming motility of the complemented strain recovered to the level of the WT (Figure 1B). Furthermore, the TEM results indicated that the lateral flagellum of ∆*VPA1701* was eliminated, while the WT and *VPA1701*^+^ were surrounded by a significant amount of lateral flagellum (Figure 1C). These results indicated that VPA1701 protein regulated the swarming motility and colonization of *V. parahaemolyticus*. 

### 2.2. The Global Transcriptional Analysis of VPA1701 in V. parahaemolyticus 

**Figure 2:**
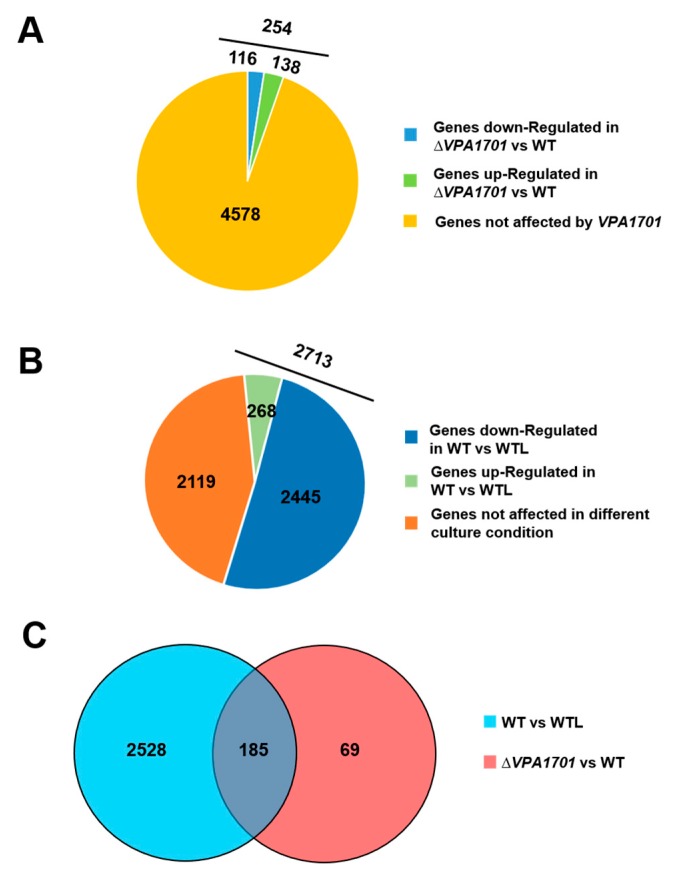
RNA-seq reveals the genes regulated by VPA1701 in the BHI agar plate. (**A**) The number of genes regulated by VPA1701. (**B**) The number of genes regulated by the WT cultured in BHI plate with 1.5% agar compared to the wild type cultured in the liquid (WTL). (**C**) Venn diagrams perform the number of genes co-regulated by VPA1701 and the BHI agar plate condition.

The previous experiment showed that the VPA1701 protein regulated the swarming motility of *V. parahaemolyticus*, and RNA-seq identified the genes regulated by VPA1701 in the BHI agar plate. The VPA1701 protein regulated 254 genes, which 116 genes were downregulated and 138 genes were upregulated (log_2_FC ≥ 2 or log_2_FC ≤ −2, *p* < 0.001) in the Δ*VPA1701* strain compared to the WT (Figure 2A). Swarming motility was analyzed in the agar plate, and the RNA-seq was also used to identify the different gene expression between the BHI agar plate and liquid medium. The results indicated that 2713 genes were regulated in the wild type cultured in agar plates (WT) compared to the wild type cultured in liquid medium (WTL) (log_2_FC ≥ 2 or log_2_FC ≤ −2, *p* < 0.001). Resultantly, 268 genes were upregulated, and 2445 genes were downregulated (Figure 2B), indicating that several genes were downregulated in the BHI agar plate. To identify the co-regulon of VPA1701 and the WT cultured in the BHI agar plates, we identified 185 overlapping genes between the regulons of VPA1701 and wild type cultured in the BHI agar plates (Figure 2C), indicating that these genes may be associated with the motility of *V. parahaemolyticus*. 

**Figure 3:**
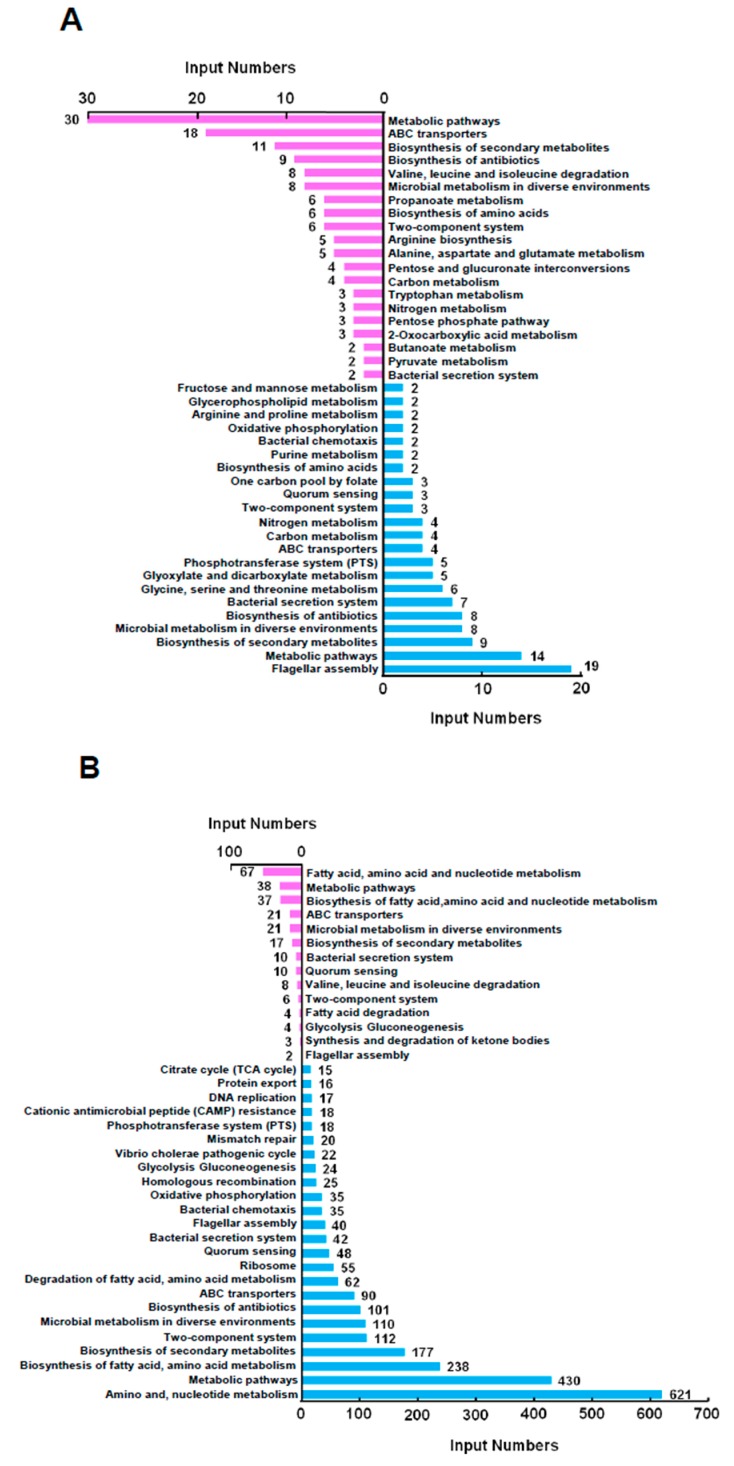
KEGG pathway analysis of the genes regulated by VPA1701 and the BHI agar plate condition. (**A**) Analysis of the gene expression in Δ*VPA1701* vs. WT. (**B**) Analysis of the gene expression in WT vs. WTL. The pink indicates the upregulated genes and the blue indicates the downregulated genes.

Based on the RNA-seq data, we performed a KEGG analysis to elucidate further the pathways regulated by VPA1701 (Figure 3A). We found that the metabolic pathways and flagellar assembly genes were altered in the Δ*VPA1701* strain. Results showed that 44 genes were involved in the metabolic pathways (30 genes were upregulated and 14 genes were downregulated), and 22 genes were involved in the ABC transporters (18 genes were upregulated and four genes were downregulated). Moreover, two-component system pathways contained three downregulated genes and six upregulated genes in the Δ*VPA1701* strain. Nineteen genes of the lateral flagellar system were downregulated in the Δ*VPA1701* compared to the WT, indicating that the regulation of swarming motility by VPA1701 was enabled by the expression of lateral flagellar genes in *V. parahaemolyticus.*

Moreover, we analyzed the KEGG pathway of the genes regulated by the WT cultured in the BHI agar plates compared to the liquid medium (Figure 3B). We found that 688 genes were involved in fatty acid, amino acid, and nucleotide metabolism pathways (67 genes were upregulated, and 621 genes were downregulated). A total of 118 genes in the two-component system were regulated in the BHI agar plate (112 genes were downregulated and six were genes upregulated). Moreover, ten genes were downregulated, and 42 genes were upregulated in the bacterial secretion system pathway. The type II secretion system and type III secretion system were downregulated, while the type VI secretion system was upregulated. Additionally, 40 polar flagellar genes that were responsible for swimming motility were downregulated in the BHI agar plate. Two lateral flagellar genes, *VPA1548* (*lafK*, the transcriptional activator) and *VPA1551* (*fliS*, a flagellar assembly gene), were upregulated. These two genes were downregulated in the Δ*VPA1701* strain, indicating that VPA1701 may regulate the expression of the lateral flagellar genes responsible for the swarming motility of *V. parahaemolyticus*.

### 2.3. Transcriptional Levels of the Lateral Flagellar Genes in ΔVPA1701

**Figure 4:**
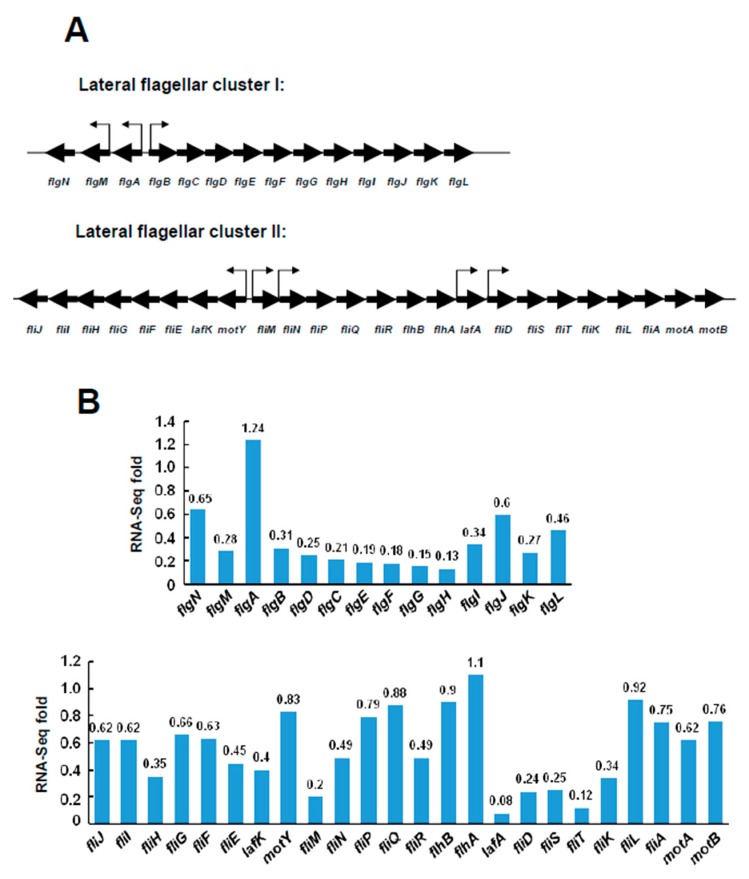
The transcription levels of lateral flagellar genes in Δ*VPA1701*. (**A**) Genetic map of the lateral flagellar cluster I and cluster II genes in *V. parahaemolyticus*. The black arrows represent the position of the predicted promoters. (**B**) Transcription levels of the two lateral flagellar genes in ∆*VPA1701* compared to the WT indicated by RNA-seq.

RNA-seq analysis indicated that the lateral flagellar genes were downregulated in the Δ*VPA1701* strain. Then, 38 genes of the lateral flagellar system were divided into two clusters, with the lateral flagellar cluster I containing 14 genes (*flgNM*, *flgA*, and *flgBCDEFGHIJKL*, from VPA0261 to VPA0274) and the lateral flagellar cluster II containing 24 genes (*fliJIHGFElafKmotY*, *fliM*, *fliNPQRflhBA*, *lafA*, and *fliDSTKLAmotAB*, from VPA1532 to VPA1557). The arrows in Figure 4A indicate the predicted promoter regions of the flagellar gene clusters. The flagellar gene clusters were downregulated by VPA1701, as shown in Figure 4B. Almost all of the genes located in the lateral flagellar cluster I were downregulated in Δ*VPA1701* compared to the WT, indicating that the expression of *flgBCDEFGHIJKL* was regulated by VPA1701 (Figure 4B). Furthermore, the *lafA*, *fliD*, *fliS*, *fliT,* and *fliK* genes of the lateral flagellar cluster II were significantly decreased in Δ*VPA1701*, suggesting that these genes were also regulated by VPA1701 (Figure 4B). Combined results of the RNA-seq analysis demonstrated that the VPA1701 protein as a transcriptional activator that regulated the expression of the lateral flagellar genes in *V. parahaemolyticus*.

### 2.4. VPA1701 Directly Binds the Promoter of VPA0264 to Activate the Expression of Lateral Flagellar Genes

**Figure 5:**
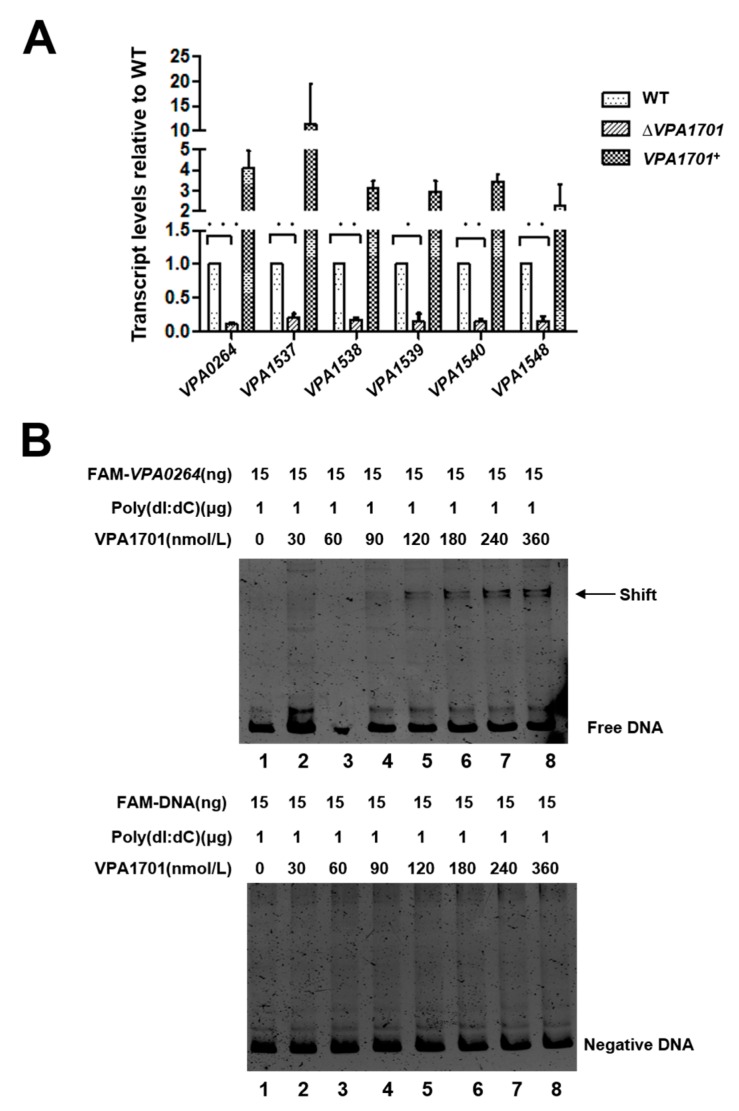
VPA1701 directly bound to the promoter of *VPA0264*. (**A**) qRT-PCR analysis of the transcription level of lateral flagellar genes (*VPA0264, VPA1537, VPA1538, VPA1539, VPA1540,* and *VPA1548*) cultured in BHI agar plates at 30 °C. The data are presented as mean ± SD (n = 3); *: *p* < 0.01; **: *p* < 0.05; ***: *p* < 0.001. (**B**) EMSAs were performed with purified VPA1701 protein, and the promoter region of the *VPA0264* was analyzed. 15 ng of FAM-labeled probes were added to the EMSA reaction and mixed with non-specific competitor DNA (poly (dI:dC)).

The RNA-seq data showed that VPA1701 significantly downregulated the lateral flagellar cluster I genes and the VPA1548-VPA1553 genes of the lateral flagellar cluster II. Thus, we selected *VPA0264,* located in the lateral flagellar cluster I, and *VPA1537*, *VPA1538*, *VPA1539*, *VPA1540,* and *VPA1548*, located in the lateral flagellar cluster II to confirm the regulation of the lateral flagellar genes by VPA170 using qRT-PCR. The expression of these six genes was significantly decreased in Δ*VPA1701*, and the complemented strain had the same transcription level as the WT (Figure 5A). Next, we used EMSA to observe the binding of VPA1701 to the promoter of lateral flagellar genes in vitro. The VPA1701 protein bound to the promoter region of *VPA0264* in a concentration-dependent manner with high concentration of a non-specific competitor. The *gyrB* probe that was used as a negative control remained unbound with the highest concentration of VPA1701 protein (Figure 5B). However, the VPA1701 could not bind to the other promoters of lateral flagellar genes (Supplementary Figure S2). In conclusion, VPA1701 protein binds directly to the promoter region of *flgBCDEFGHIJKL* to activate the expression of the lateral flagellar system that controls the swarming motility of *V. parahaemolyticus*. 

### 2.5. Calcium Restored the Swarming Ability of ∆VPA1701

**Figure 6:**
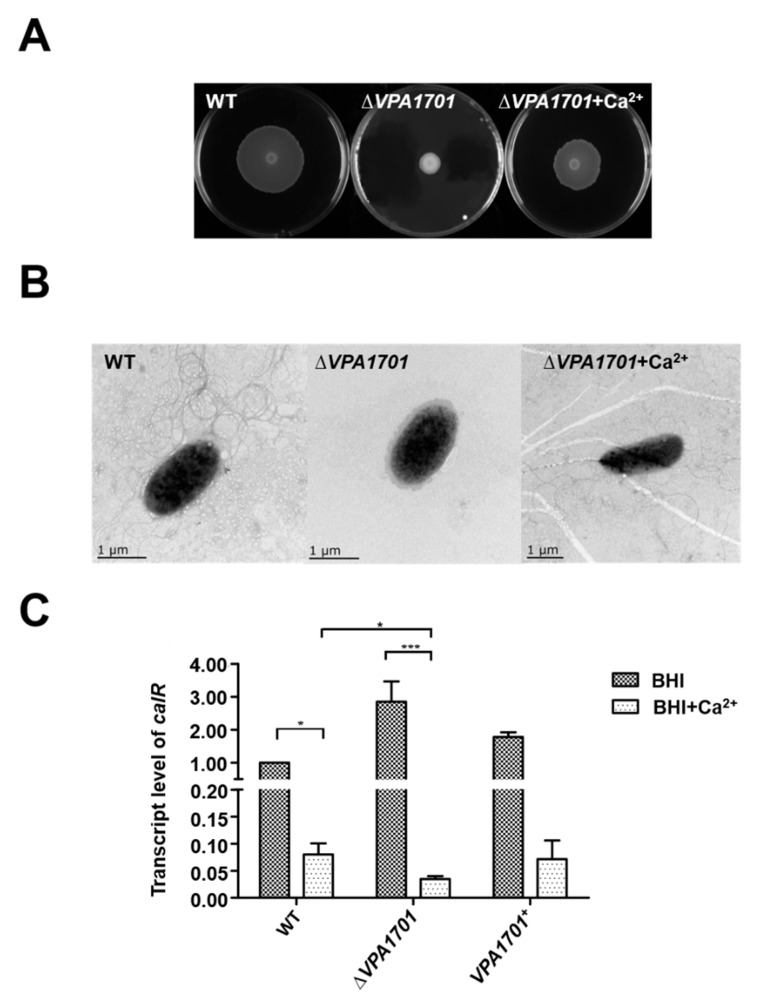
Calcium restores the swarming motility of ∆*VPA1701*. (**A**) The swarming motility analysis of WT and ∆*VPA1701* in the BHI agar plates, with and without 5 mM calcium. (**B**) The generation of lateral flagella in the WT and ∆*VPA1701* cultured in the BHI agar plates, with and without 5 mM, calcium was observed by TEM. (**C**) The transcription levels of *calR* in WT and ∆*VPA1701* cultured in the BHI agar plates, with and without 5mM calcium, was assessed using qRT-PCR. The data are presented as mean ± SD (n = 3); *: *p* < 0.01; **: *p* < 0.05; ***: *p* < 0.001.

CalR is a LysR-type transcriptional regulator that was inhibited by the Calcium in *V. parahaemolyticus* [25]. CalR was originally identified as a repressor of the swarming motility, TDH2, and T3SS1, and previous studies have demonstrated that the biofilm formation, hemolytic activity and T6SS2 also could be regulated by CalR in *V. parahaemolyticus* [25,33,34,35]. When the ∆*VPA1701* strain was cultured in the BHI agar plates with 5 mM calcium, the swarming motility of ∆*VPA1701* was recovered, but the swarming level was lower than the WT (Figure 6A). This may have been due to the decreased expression of lateral flagellar genes in the absence of VPA1701. TEM analysis further indicated that calcium restored the generation of the lateral flagellum of ∆*VPA1701* (Figure 6B). In addition, we used qRT-PCR to detect the expression level of *calR* in WT, ∆*VPA1701,* and *VPA1701*^+^ in the BHI agar plates, with and without 5 mM calcium. The transcription level of *calR* significantly increased in ∆*VPA1701* compared to the WT without calcium, and it decreased by 50% with 5 mM calcium (Figure 6C), indicating that the inhibition of calcium to *calR* had a greater influence on the expression of *calR* than VPA1701. Furthermore, the expression of *calR* was significantly decreased in ∆*VPA1701* with 5 mM calcium compared to the ∆*VPA1701* mutant strain without calcium (Figure 6C). Together, these results demonstrated that VPA1701 inhibited the expression of *calR* to regulate the swarming motility of *V. parahaemolyticus*, and calcium compensated for the inhibition of *calR* by VPA1701 and restored the swarming motility of ∆*VPA1701*.

**Figure 7:**
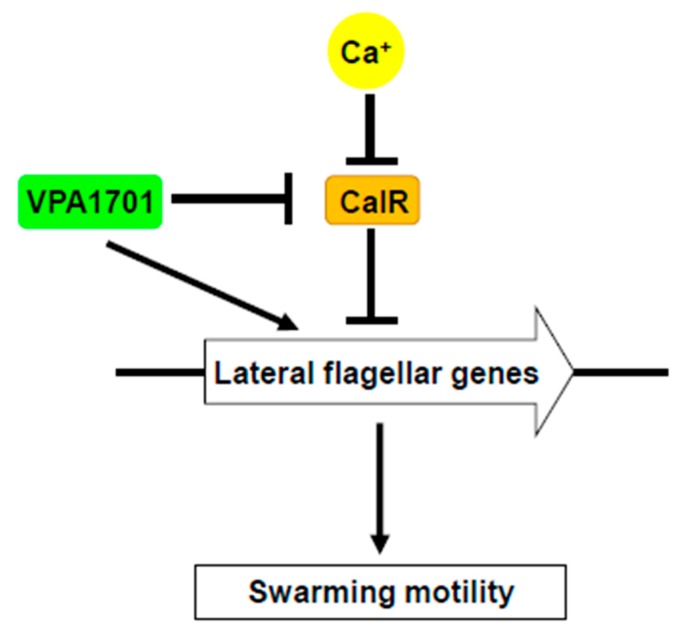
The regulatory network of VPA1701 for swarming motility in *V. parahaemolyticus* VPA1701 signaling increases the expression of lateral flagellar genes and inhibits the transcription of the *calR* gene to control the swarming motility of *V. parahaemolyticus*. Calcium and VPA1701 inhibit the expression of *calR* and de-repression of CalR to the lateral flagellar genes to control the swarming motility.

Based on these results, we propose a model for the regulation of the swarming motility by VPA1701 in *V. parahaemolyticus* (Figure 7). VPA1701 is a transcriptional regulator in the GntR family that specifically binds to the *flgBCDEFGHIJKL* promoter region to regulate the expression of lateral flagellar genes that are responsible for the swarming motility. Moreover, VPA1701 also inhibits the transcription of *calR* and promotes the expression of lateral flagellar genes in *V. parahaemolyticus*. In the presence of calcium, the expression of *calR* is inhibited, and the swarming motility of ∆*VPA1701* is restored. These data facilitate an improved understanding of the regulatory networks of the transcription factor VPA1701 contributing to the swarming motility and colonization of *V. parahaemolyticus*.

## 3. Materials and Methods

### 3.1. Bacterial Strains, Plasmids and Culture Conditions

*V. parahaemolyticus* RIMD 2210633 wild-type (WT) and its deviants (mutants and complements), as well as E. coli, were grown in Luria-Bertani (LB) broth at 37 °C, shaken at 200 rpm/min. The swarming motility analysis of *V. parahaemolyticus* strains were cultured in Brain Heart Infusion (BHI) with 1.5% agar. When necessary, suitable antibiotics were added: carbenicillin (Carb, 50 μg/mL), chloramphenicol (Cm, 25 μg/mL), and kanamycin (Km, 50 μg/mL). Detailed information on the bacterial strains, plasmids, and primers used in this study is listed in Supplementary Tables S1 and S2. 

### 3.2. Construction of the VPA1701 Mutant and Complemented Strains 

An in-frame deletion mutant of *V. parahaemolyticus* RIMD 2210633 *VPA1701* was constructed as previously described in Reference [36]. The upstream fragment (643 bp) and downstream fragment (764 bp) of *VPA1701* were amplified by PCR with specific primers (Supplementary Table S2) and connected by overlap PCR. The overlap fragments (1407 bp) were cloned into the suicide vector pDM4 with Sac I/Xho I sites and then transformed into *E. coli* DH5α *λpir* and *E. coli* SM10 *λpir*. The recombinant plasmid *VPA1701*::pDM4 was transformed into the wild type by conjugation, and the conjugant was plated in LB agar with Carb and Cm. Then, the second cross-over recombination was placed in LB agar containing 10% sucrose. The mutant strain (∆*VPA1701*) was verified by PCR (*VPA1701*-out/in-F/R) and sequencing. 

To construct the VPA1701 complemented strain, the RBS and ORF regions of VPA1701 were amplified by PCR with specific primers (VPA1701-F/R) and then cloned into the pMMB207 plasmid with Sal I/Sac I sites. Then, the recombinant plasmid (*VPA1701*::pMMB207) was transformed into *E. coli* SM10 *λpir* and conjugated to the ∆*VPA1701* strain. The conjugants were placed in LB agar containing Carb and Cm and confirmed by PCR (pMMB207-F/R) and sequencing. When necessary, 0.1 mM IPTG was added to induce the expression of *VPA1701*. The complemented strain was defined as *VPA1701^+^.*

### 3.3. Motility Analysis

The swimming and swarming motility assays were set up as previously described in Reference [37]. WT, ∆*VPA1701,* and *VPA1701^+^* strains were cultured overnight and diluted to 1:100 in new LB broth. Then, 2 μL of diluted cultures (OD_600_ = 1.0) were spotted on the LB plates containing 0.3% agar at 37 °C for 12 h and BHI plates containing 1.5% agar at 30 °C for 24 h to investigate the swimming and swarming motility separately. All the experiments were conducted three times.

### 3.4. Transmission Electron Microscope (TEM) of the Lateral Flagellar

The WT, ∆*VPA1701,* and *VPA1701^+^* strains were incubated in BHI agar for 15 h, washed gently with 0.01 M PBS, and then placed into separate 2 mL tubes. Subsequently, 5 μL of bacterial suspensions were dripped onto the copper grid with a supporting membrane and they were left for 5 min until the mesh was dry enough to cover. Then, 5% uranyl acetate was used to cover the dry samples for 30 min in a dry environment and observed by TEM (JEM 2100, Tokyo, Japan). 

### 3.5. Quantification Real-Time Reverse Transcription PCR (qRT-PCR)

The *V. parahaemolyticus* WT, ∆*VPA1701*, and *VPA1701^+^* strains were cultured in BHI agar plates for 15 h, and then the cell cultures were harvested from the plates. The total RNA was extracted using the Bacterial Total RNA Extraction Kit (Shangon Biotech, Shanghai, China) and treated with RNase-free DNase I to remove the contamination of genomic DNA. Then, 1 μg of total RNA was used to generate the first strand of cDNA by the reverse transcriptase (Takara, Tsuruga, Japan). The qRT-PCR was performed by the specific primers listed in the Supplementary Table S2, using an ABI StepOnePlus Real-Time PCR System with SYBR^®^ Premix Ex TaqTM (Takara, Tsuruga, Japan). The transcription levels were normalized to the *gyrB* in each sample using the 2^−ΔΔCt^ method, as described in Reference [38].

### 3.6. RNA-Seq Analysis 

The *V. parahaemolyticus* WT and ∆*VPA1701* strains were cultured in BHI agar plates for 15 h, the wild type was cultured in LB broth for 9 h, and then the bacterial cells were collected. Total RNA was extracted using the Bacterial Total RNA Extraction Kit (Shangon Biotech, Shanghai, China). RNA samples were digested with DNase I (Promega, Madison, WI, USA) to eliminate genomic DNA contamination. Three parallel RNA samples of each strain were sequenced using the illumina HiSeq (GENEWIZ, Suzhou, China). The subsequent procedures and statistical analysis were conducted as previously described in Reference [39].

### 3.7. Overexpression and Purification of VPA1701 Protein

The ORF region of the VPA1701 was amplified by PCR with specific primers (Supplementary Table S2) and then cloned into the pET30a plasmid. The recombinant plasmid (*VPA1701*::pET30a) was transformed into *E. coli* BL21(DE3), cultured in LB plates containing Carb to select the positive clone with the primers (pET30a-F/R), and then confirmed by sequencing. 

The bacteria *VPA1701*::pET30a/BL21 was cultured overnight, diluted to 1% in 200 mL of fresh LB broth, and IPTG was added to induce the expression of protein until the OD_600_ was between 0.4–0.6. Then this was cultured at 22 °C for 12 h, shaken at 120 rpm/min. The cell cultures were collected, washed, and resuspended. The Ni^2+^ column purified the His-tagged VPA1701 protein. The VPA1701 protein was verified by 12% SDS-PAGE, and the concentration was determined using a nucleic acid analyzer (Nano-200, Shanghai, China). 

### 3.8. Electrophoretic Mobility Shift Assay (EMSA) 

The EMSA analysis was performed as previously described in Reference [38]. The DNA probes were amplified by the specific primers using a FAM fluorescent label (Supplementary Table S2) and purified using a DNA Gel Purification Kit. The concentration of the DNA probes was measured using the nucleic acid analyzer. The EMSA reaction (20 μL) was mixed with 15 ng of DNA probes, 1 μg of Poly-dIdC, and 4 μL of 5× Binding buffer (10 mM NaCl, 0.1 mM DTT, 0.1 mM EDTA, 10 mM Tris, pH 7.4), thereby increasing the amount of VPA1701-his protein. Then it was incubated at 25 °C for 30 min, where 2 μL of 10× EMSA/Gel shift loading buffer was added to each sample, and it was separated by a 6% native-PAGE gel in 0.5× TBE buffer (54 mM Tris-base, 27.5 mM Boric acid, 4.12 mM EDTA) for 2 h with 100 V. The gel was scanned using a Typhoon FLA 9500 (GE healthcare, Uppsala, Sweden). The *gyrB* promoter was used as a negative control. 

### 3.9. Infant Rabbit Model of V. parahaemolyticus Infection

Infant rabbits were used to observe the intestinal colonization of *V. parahaemolyticus* [40,41]. The overnight cultured WT and ∆*VPA1701* were placed in fresh LB medium at 30 °C for 18 h. Then, 2 mL of each sample was collected and washed using PBS three times, and then resuspended with a 4 ml 2.5% NaHCO_3_ solution for each sample. Three-day-old rabbits received 100 μL of bacterial suspension, delivered orogastrically, and they were observed for diarrhea and death for 36 h. Then the rabbits were euthanized and dissected, and 0.1 g of intestinal tissue was removed, smashed, and suspended with 1 mL of PBS. Then this was diluted to 10^−1^–10^−6^ and plated in LB agar containing Carb to determine the colonization of *V. parahaemolyticus*. All animal experiments of this study were approved by the institutional administrative committee and ethics committee of laboratory animals (Approval No. SYXK 2016-0019) and conducted following the guidelines approved by the Animal Welfare and Ethics Committees of Yangzhou University.

## Supplementary Materials

The following are available online at https://www.mdpi.com/2076-0817/8/4/235/s1. Table S1: Bacterial strains and plasmids used in this study. Table S2: Primers used in this study. Figure S1: Multiple amino acid sequence alignments of VPA1701 proteins in Vibrios. The amino acid sequence alignments of VPA1701 protein among *V. parahaemolyticus* and the homology proteins in *Vibrio harveyi*, *Vibrio campbellii*, *Vibrio vulnificus* and *Vibrio cholerae*. The identical and highly conserved amino acid sequence were indicated by red rectangular frames. Figure S2: No specific binding of VPA1701 and the promoter regions of VPA1548 and VPA1551. EMSA assay using purified VPA1701 protein and VPA1548 and VPA1551 probes with the increasing amount of VPA1701 protein.
